# Deep learning reveals antibiotics in the archaeal proteome

**DOI:** 10.1038/s41564-025-02061-0

**Published:** 2025-08-12

**Authors:** Marcelo D. T. Torres, Fangping Wan, Cesar de la Fuente-Nunez

**Affiliations:** 1 Machine Biology Group, Departments of Psychiatry and Microbiology, Institute for Biomedical Informatics, Institute for Translational Medicine and Therapeutics, Perelman School of Medicine, University of Pennsylvania, Philadelphia, PA USA; 2 Departments of Bioengineering and Chemical and Biomolecular Engineering, School of Engineering and Applied Science, University of Pennsylvania, Philadelphia, PA USA; 3 Department of Chemistry, School of Arts and Sciences, University of Pennsylvania, Philadelphia, PA USA; 4 Penn Institute for Computational Science, University of Pennsylvania, Philadelphia, PA USA

**Keywords:** Microbiology, Antimicrobials

## Abstract

Antimicrobial resistance is one of the greatest threats facing humanity, making the need for new antibiotics more critical than ever. While most antibiotics originate from bacteria and fungi, archaea offer a largely untapped reservoir for antibiotic discovery. In this study, we leveraged deep learning to systematically explore the archaeome, uncovering promising candidates for combating antimicrobial resistance. By mining 233 archaeal proteomes, we identified 12,623 molecules with potential antimicrobial activity. These peptide compounds, termed archaeasins, have unique compositional features that differentiate them from traditional antimicrobial peptides, including a distinct amino acid profile. We synthesized 80 archaeasins, 93% of which showed antimicrobial activity in vitro against *Acinetobacter baumannii*, *Escherichia coli*, *Klebsiella pneumoniae*, *Pseudomonas aeruginosa*, *Staphylococcus aureus* and *Enterococcus* spp. Notably, in vivo validation identified archaeasin-73 as a lead candidate, significantly reducing *A. baumannii* loads in mouse infection models, with effectiveness comparable to that of established antibiotics such as polymyxin B. Our findings highlight the potential of archaea as a resource for developing next-generation antibiotics.

## Abstract (web summary)

Use of artificial intelligence to mine proteomes of archaea led to the discovery of archaeasins, antimicrobials that kill drug-resistant bacteria in laboratory and animal models, offering a promising source of future antibiotics.

## Main

The rise of antimicrobial resistance is one of the most urgent global health threats, as resistant pathogens undermine the efficacy of existing antibiotics, leading to increasingly difficult-to-treat infections. This growing crisis highlights the critical need for new antibiotics^1^. However, the discovery pipeline for antibiotics has slowed substantially in recent decades^1,2^, and has traditionally relied primarily on bacteria and fungi as sources. Recently, computational approaches^3–7^, particularly deep learning models, have provided new avenues for antibiotic discovery by enabling the systematic exploration of vast sequence spaces.

Despite their evolutionary significance and biochemical diversity, archaea remain an underexplored domain for antibiotic discovery. Unlike bacteria and eukaryotes, archaea possess unique lipid membranes, metabolic pathways and stress-adaptation mechanisms that may influence the structure and function of their encrypted peptides (EPs). EPs have emerged as an exciting new frontier in antibiotic discovery due to their unique structural and functional properties. These peptides are often overlooked in conventional sequence-based searches but can show broad-spectrum antimicrobial activity when properly identified and tested. Previous studies have shown that EPs derived from human^4,7^, bacterial^3,8^ and even extinct organisms^5,6^ proteomes can serve as effective antimicrobial agents, yet no systematic investigation has explored the archaeome for such bioactive sequences. Given their evolutionary divergence from bacteria and eukaryotes, archaeal EPs may have structural and mechanistic properties that differentiate them from known antimicrobial agents.

**Extended Data Fig. 1:**
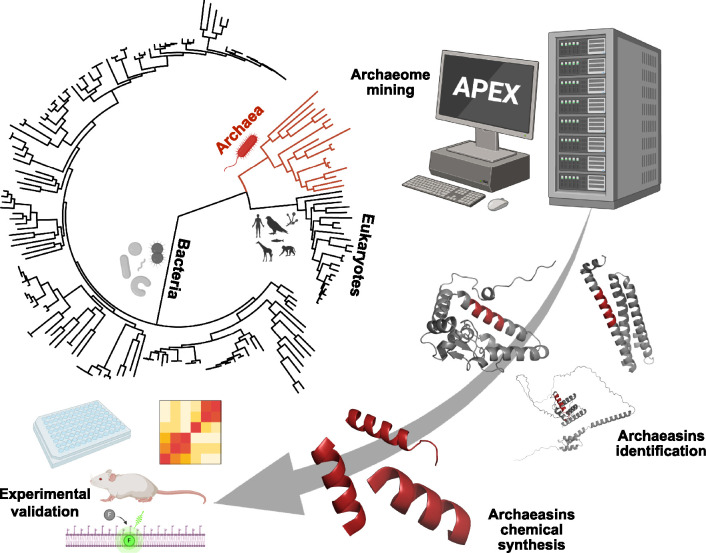
Discovery of antibiotics from Archaea using deep learning. The archaeome was systematically mined using our deep learning algorithm, APEX. Peptide sequences ranging from 8 to 50 amino acid residues within archaeal proteins were analyzed through multitask deep learning models trained on both public and in-house peptide datasets to predict antimicrobial activity. The top-ranked peptides, based on their predicted antimicrobial potential, were chemically synthesized and extensively evaluated against clinically relevant pathogens in both *in vitro* and animal model studies. Comprehensive assays were conducted to investigate the mechanism of action, toxicity, physicochemical properties, and potential synergistic interactions of these peptides. The protein and peptide structures depicted in the figure were created with PyMOL Molecular Graphics System, version 3.0 Schrödinger, LLC. Figure created with BioRender.com.

In this study, we applied APEX 1.1, an updated version of our previously developed deep learning framework, APEX^6^, to systematically mine all archaeal proteomes curated from the Swiss-Prot^9^ subset of UniProt^10^. This approach enabled the identification of EPs with predicted antimicrobial activity, herein referred to as archaeasins (Extended Data Fig. 1). By leveraging a computational pipeline trained on known antimicrobial peptides (AMPs) and EP sequences, we identified 12,623 putative AMPs from 233 archaeal proteomes. Of these, we synthesized and experimentally tested 80 archaeasins, with 93% showing antimicrobial activity in vitro. Furthermore, we validated the efficacy of archaeasin-73 in preclinical murine infection models, where it showed antimicrobial effects comparable to those of polymyxin B.

By expanding the search for encrypted AMPs into the archaeome, this work highlights the untapped potential of this domain of life and underscores the power of integrating deep learning with experimental validation to accelerate antibiotic discovery. Our findings provide a large-scale demonstration that archaea encode a vast repertoire of peptides with promising therapeutic potential.

## Results

### Deep-learning-guided identification of archaeasins

**Fig. 1:**
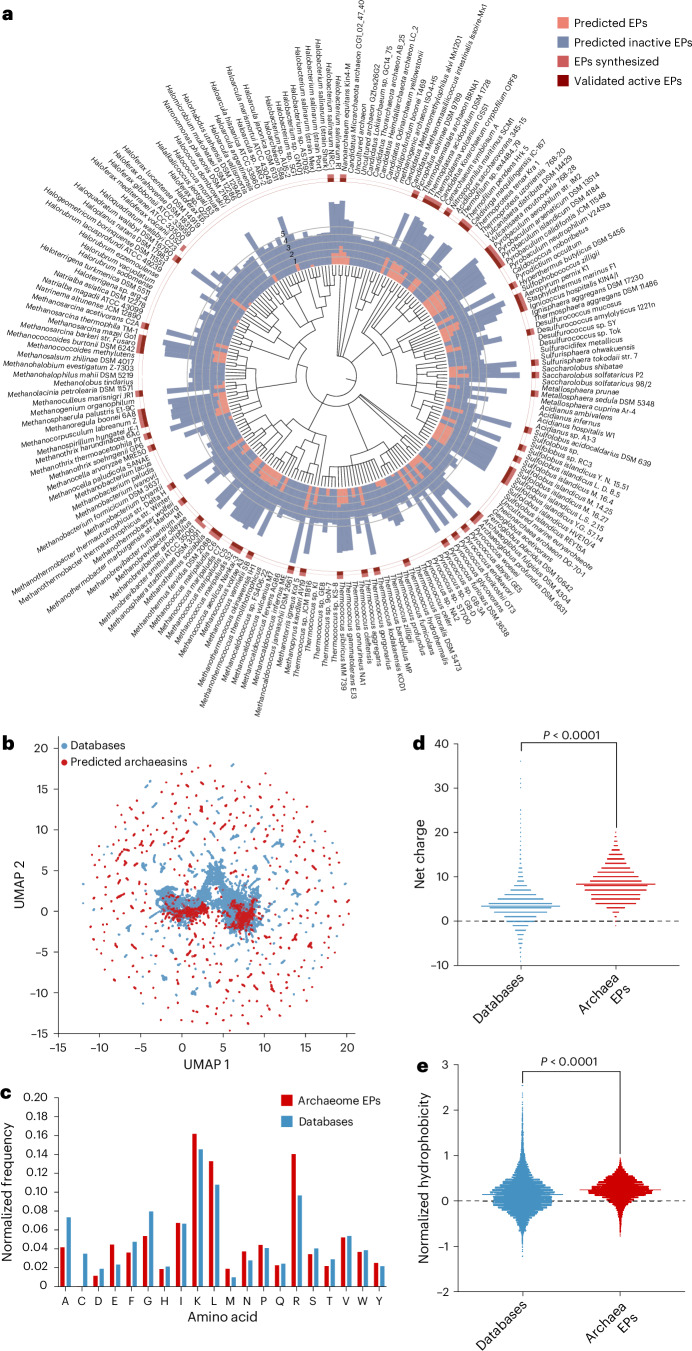
APEX exploration of the archaeome. **a**, Archaeal proteomes were systematically scanned to identify EPs with potential antimicrobial activity. Circular bars denote the log_10_-transformed average active (red) and inactive (blue) EPs discovered by APEX. A peptide was classified as active if its predicted mean MIC against tested bacterial strains was ≤100 μmol l^−1^. The values were normalized by the number of proteins per organism scanned. Archaea with peptides that were synthesized are indicated by a light red square, and those experimentally validated as active are highlighted with a dark red square. **b**, Sequence space exploration using a similarity matrix. The graph illustrates a bidimensional sequence space visualization of peptide sequences found in DBAASP and antimicrobial EPs discovered by APEX in archaea organisms. Sequence alignment was used to generate a similarity matrix for all peptide sequences in DBAASP and the 12,623 antimicrobial EPs predicted by APEX (Supplementary Data 1 and 2). Each row in the matrix represents a feature representation of a peptide based on its amino acid composition. UMAP was applied to reduce the feature representation to two dimensions for visualization (Extended Data Fig. 2a). **c**, Comparison of amino acid frequency in archaeal EPs with known AMPs from the DBAASP, APD3 and DRAMP 3.0 databases (Extended Data Fig. 2b–e). **d**,**e**, Distribution of two physico-chemical properties for peptides with predicted antimicrobial activity, compared with AMPs from DBAASP, APD3 and DRAMP 3.0: net charge (**d**) and normalized hydrophobicity (**e**). Net charge influences the initial electrostatic interactions between the peptide and negatively charged bacterial membranes, whereas hydrophobicity affects interactions with lipids in the membrane bilayers (Extended Data Fig. 2). The Chi-squared test of independence of variables in a contingency table was used to compare the amino acid composition in **c**; *P* values were 0, that is, below machine precision levels, suggesting that they are statistically significant. Statistical significance in **d** and **e** was determined using two-tailed *t*-tests followed by a Mann–Whitney test; *P* < 0.0001. The solid line inside each box represents the mean value for each group. Source data

We collected 18,677 non-redundant reviewed protein sequences from 233 archaeal organisms available on UniProt^10^ and used APEX 1.1, a deep learning antimicrobial activity predictor^6^ retrained on updated data (‘APEX 1.1’ in Methods), to mine EPs within archaeal proteomes. As APEX predicted bacterial-strain-specific minimum inhibitory concentrations (MICs), we used the mean MIC to represent the overall antimicrobial potency of the peptides and found 12,623 EPs with a mean MIC ≤100 μmol l^−1^ (Fig. 1a and Supplementary Data 1), representing an archaeasin ratio of 0.00653% from the 193,331,608 archaeal peptides scanned. To assess whether this signal exceeded random expectations, we generated a non-redundant set of 193,288,387 randomly sampled peptides with a length distribution matching that of the archaeasins. Applying the same AMP criterion (mean MIC ≤ 100 μmol l^−1^), we identified 5,292 predicted active peptides from the random set, corresponding to an AMP rate of 0.00274%, or roughly 2.38× lower than that observed in archaeal EPs. These findings suggest that antimicrobial sequences are statistically enriched in archaeal proteomes relative to random sampling.

We next examined whether archaeal species with larger genomes tend to encode a greater number of predicted AMPs. To do this, we compiled the number of predicted active EPs (defined as peptides with a mean MIC ≤100 μmol l^−1^ across 11 pathogen strains) and the total number of protein-coding genes, based on National Center for Biotechnology Information (NCBI) annotations, for the 10 archaeal genera with the highest and lowest EP counts. This dataset enabled us to assess potential relationships between genomic content and AMP abundance (Supplementary Table 1). A Spearman correlation analysis revealed a statistically significant positive correlation between genome size and the number of predicted EPs (Spearman’s rank correlation coefficient (*ρ*) = 0.4475, *P* = 0.0479), suggesting that archaeal species with larger genomes may harbour a broader repertoire of latent antimicrobial sequences.

Interestingly, several of the top-scoring genera, such as *Pyrococcus*, *Methanocaldococcus*, *Pyrobaculum* and *Sulfolobus*, are known thermophiles. This observation raises the possibility that certain lifestyle traits, such as adaptation to high-temperature environments, may be associated with greater bioactive peptide abundance. Although this analysis focused primarily on genome size, we acknowledge that environmental factors such as temperature tolerance, metabolic specialization or ecological niche may also influence the diversity and prevalence of encrypted AMPs. However, we consider this a preliminary analysis and expect that, as more archaeal genomes are catalogued and annotated, the observed trends will become more robust and enable more detailed exploration of ecological and evolutionary correlates of active peptide abundance.

**Extended Data Fig. 2:**
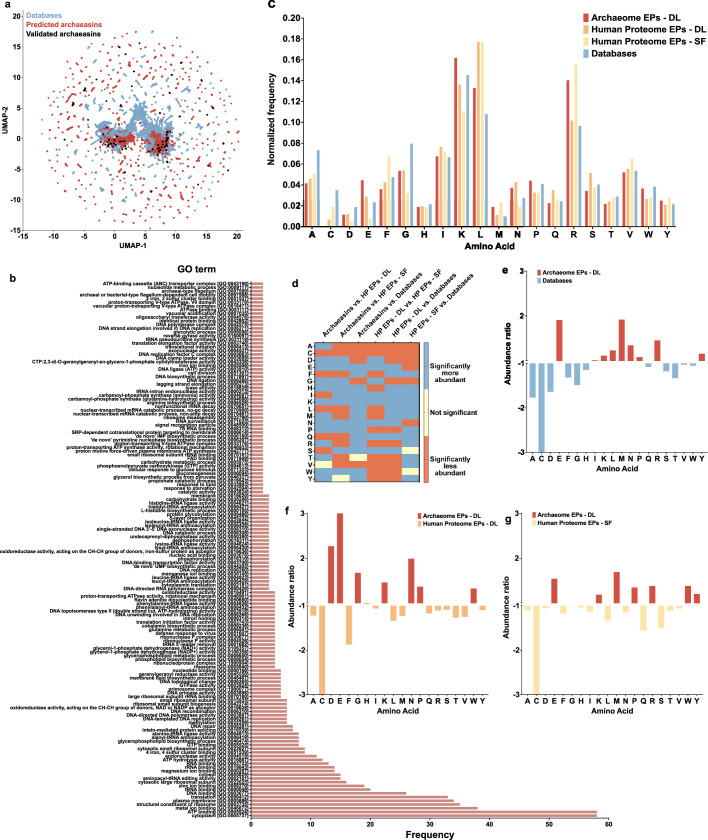
Sequence features of the archaeasins. **(a)** Bidimensional sequence space visualization of peptide sequences found in DBAASP, all predicted antimicrobial EPs discovered by APEX, and peptides that were synthesized and validated in this study. Uniform Manifold Approximation and Projection (UMAP) was used to reduce the feature representation to two dimensions for visualization purposes. **(b)** Frequency of the gene ontology annotations of the top 265 archaeasins with mean MIC < 80 μmol L^−1^. **(c)** Amino acid frequency in archaeasins compared with known AMPs from DBAASP, APD3, and DRAMP 3.0 databases, and other encrypted peptides from the human proteome discovered by APEX^6^ and a scoring function^4^. **(d)** To further explore differences at the individual residue level, we calculated the percentage of each amino acid residue within every peptide sequence and used a one-sided Mann-Whitney U test to compare these percentages between peptide sets. A significance threshold of p < 0.01 was used to identify amino acids that were significantly more or less abundant between specific sets. The exact p-values for each comparison are shown in Supplementary Data 3 and 4 files (under Data availability). **(e-g)** Relative abundance of the amino acid content of Archaea encrypted peptides identified by APEX (red) and **(d)** AMPs from the DBAASP, APD3, and DRAMP 3.0 database (blue), **(e)** human proteome encrypted peptides discovered by APEX (orange), and **(f)** human encrypted peptides found by the scoring function (yellow). The frequency of amino acid was normalized by the total number of amino acid residue counts. Chi-square test of independence of variables in a contingency table was used to compare the amino acid composition in **c-f**, p values were 0, that is, below machine precision levels suggesting that they are statistically significant. Source data

To investigate the distribution of AMP-like EPs within the archaeal proteomes, we first performed sequence alignment on the combined set of 12,623 EPs and 19,775 publicly available AMPs from DBAASP^11^, APD3 (ref. ^12^) and DRAMP^13^ (see ‘APEX 1.1’ in Methods). We then applied uniform manifold approximation and projection (UMAP)^14^ to reduce and visualize the sequence similarity matrix derived from the local sequence alignment (Fig. 1b and Extended Data Fig. 2a).

Focusing on the top 265 archaeasins (mean MIC < 80 μmol l^−1^; Supplementary Data 2) that are sequentially diverse (see ‘Archaeasin selection’ in Methods), we analysed their source proteins by retrieving Gene Ontology annotations (Extended Data Fig. 2b). Gene Ontology term frequencies revealed that many of these top-ranking archaeasins originated from cytoplasmic proteins and proteins with essential cellular functions, including ATP binding, metal-ion binding, DNA binding, tRNA binding and zinc-ion binding. Several archaeasins were also derived from structural ribosomal proteins, plasma membrane proteins and proteins involved in translation. Collectively, these findings highlight the broad distribution and functional diversity of archaeasins throughout archaeal cells.

**Extended Data Fig. 3:**
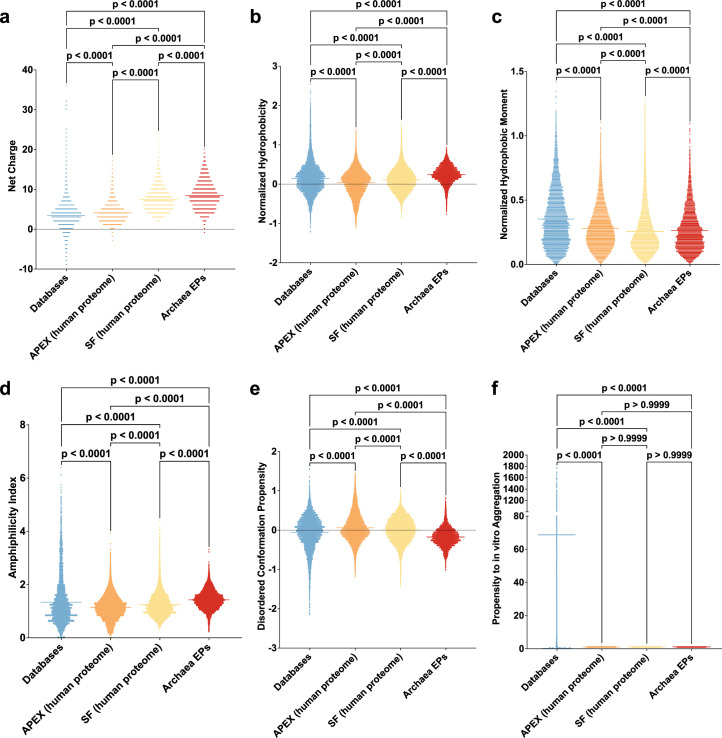
Physicochemical features of archaeasins compared to AMPs from databases (DBAASP, APD3, and DRAMP 3.0), and encrypted peptides from human proteins identified by APEX and a scoring function. **(a)** Net charge and **(b)** normalized hydrophobicity. **(c)** Hydrophobic moment normalized by peptide length, reflecting the amphipathicity of the molecules, which directly influences their interactions with bacterial membranes. **(d)** Amphiphilicity index and **(e)** disordered conformation propensity, both of which are closely correlated with the mechanism of action, specifically how peptides interact with membrane lipids to exert antimicrobial activity. **(f)** Propensity to aggregate *in vitro*, correlated with the supramolecular arrangement of the molecules and potential toxicity. Statistical significance was determined using two-tailed t-tests followed by the Mann-Whitney test; p values are shown in the graph. The solid line within each box represents the mean value for each group. Source data

The amino acid composition of archaeasins revealed distinctive features compared with known AMPs from databases (Fig. 1c) and EPs previously discovered in the human proteome using APEX^6^ and a complementary scoring function^4,7^ (Extended Data Fig. 2c–f). Archaeasins were notably enriched in glutamic acid residues, surpassing levels typically found in known AMPs. This higher prevalence of negatively charged residues is also observed when comparing archaeasins with other EPs from human proteins. Nonetheless, archaeasins maintain a prevalence of cationic residues, leading them to display a slightly higher proportion of cationic residues compared with database entries, suggesting a unique balance in charge distribution (Fig. 1d). Despite these differences, their hydrophobicity remains comparable to standard database sequences (Fig. 1e). In addition, archaeasins show a tendency towards increased amphiphilicity, indicating a balanced distribution between hydrophobic and hydrophilic residues (Extended Data Fig. 3 and Supplementary Table 2). This analysis supports the notion that archaea, like humans^7^, encode a rich and compositionally unique repertoire of antimicrobial EPs, highlighting their potential as an unusual source of antibiotics and providing an evolutionary contrast that aids in deciphering antimicrobial diversity across domains of life.

### Antimicrobial activity of archaeasins against bacterial pathogens

To experimentally validate the antimicrobial activity of the archaea EPs, we selected 80 peptides that were both sequentially diverse (<70% sequence similarity with each other) and top ranked by APEX 1.1 (Supplementary Data 2). We prioritized peptides with less than <70% sequence similarity to known AMP sequences for chemical synthesis and experimental validation (Extended Data Fig. 2a). In addition, when two mined sequences showed high sequence similarity, we retained only the peptide with the higher predicted antimicrobial activity (see ‘Archaeasin selection’ in Methods).

**Fig. 2:**
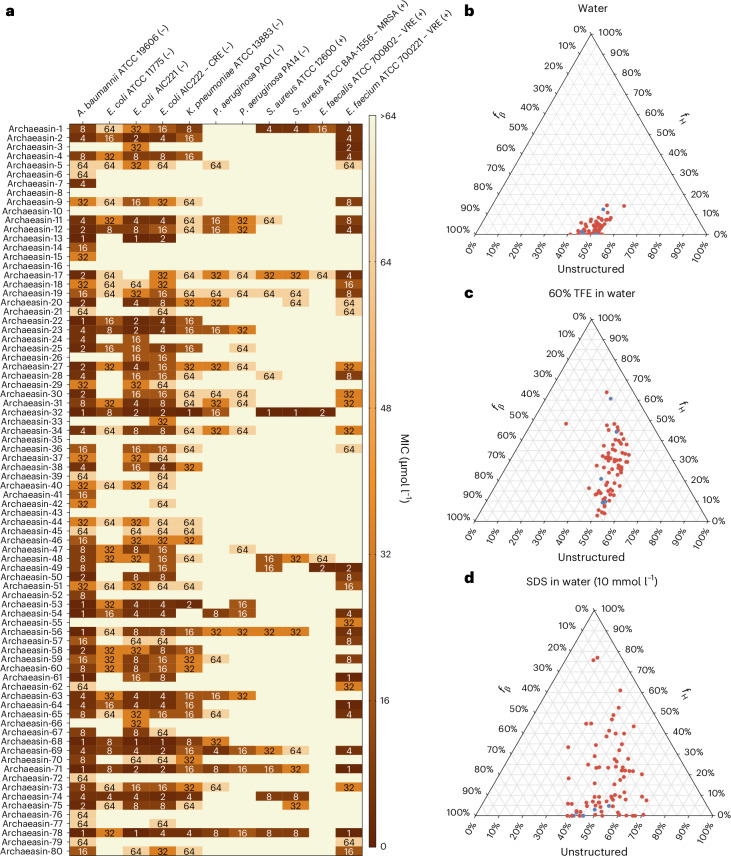
Antimicrobial activity and secondary structure profiles of antibiotics from the archaeome. **a**, Heat map showing the antimicrobial activities (μmol l^−1^) of active antimicrobial agents from archaea against 11 clinically relevant pathogens, including Gram-negative (indicated by –) and Gram-positive (indicated by +) antibiotic-resistant strains (CRE, colistin-resistant *Escherichia coli*; MRSA, methicillin-resistant *Staphylococcus aureus*; VRE, vancomycin-resistant *Enterococci*). Briefly, 10^5^ bacterial cells were incubated with serially diluted EPs (0–64 μmol l^−1^) at 37 °C. Bacterial growth was assessed by measuring the optical density at 600 nm in a microplate reader at 1 day post-treatment. The MIC values presented in the heat map represent the mode of the replicates for each condition, and the antibiotics polymyxin B and levofloxacin were used as positive controls (Extended Data Fig. 4a). **b−d**, Ternary plots showing the percentage of secondary structure for each peptide (at 50 μmol l^−1^) in 3 different solvents: water (**b**), 60% TFE in water (**c**), and SDS (10 mmol l^−1^) in water (**d**). Secondary structure fractions were calculated using the BeStSel server^18^. *f*_H_ and *f*_β_ stand for helical and β fractions, respectively. Red dots indicate active archaeasins and blue dots represent inactive peptides (Extended Data Fig. 5). Source data

**Extended Data Fig. 4:**
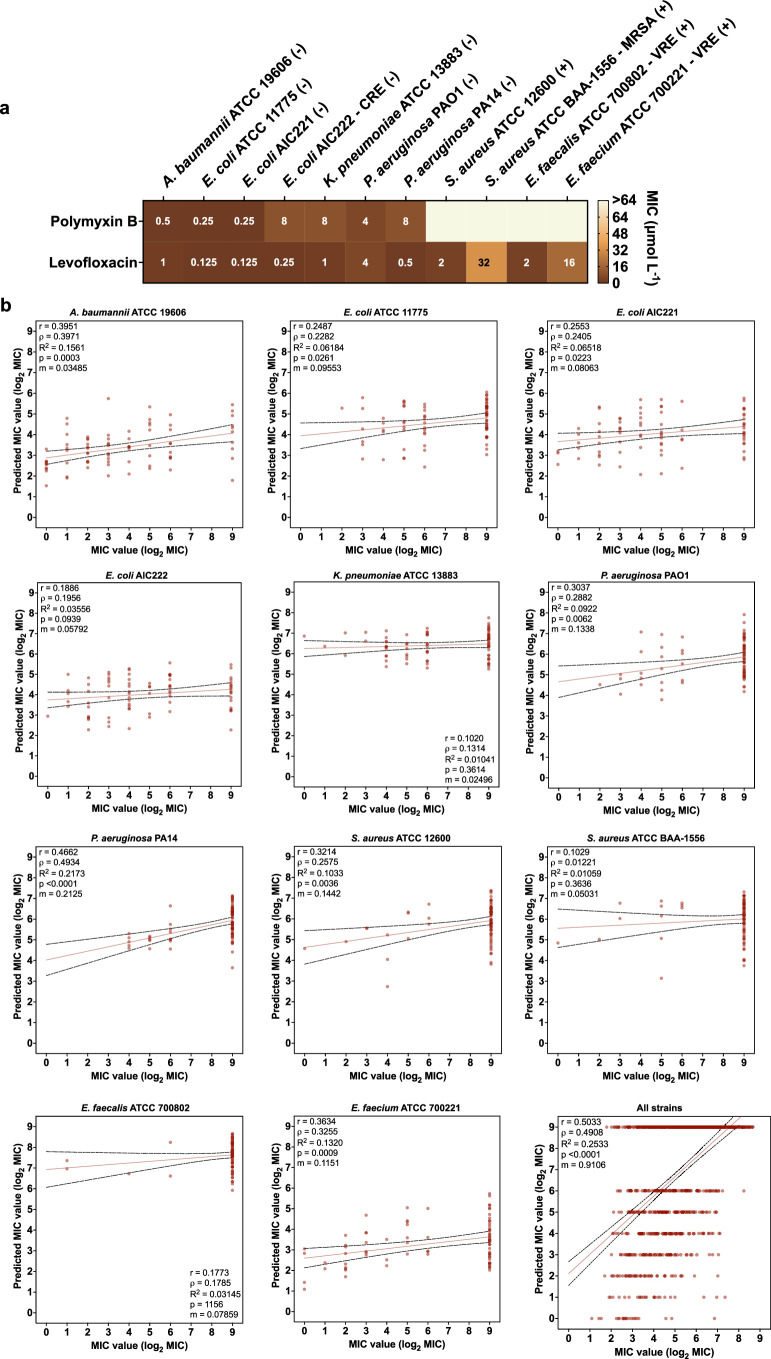
Control antibiotics and predicted vs. experimental MIC values of the archaeasins identified by APEX against various pathogens. **(a)** Heatmap showing the activity of the antibiotics polymyxin B and levofloxacin used as controls in our experiments. The results represent the mode of three independent replicates. **(b)** Each peptide is represented by a red circle on the scatter plot. The inset includes Pearson (r) and Spearman (ρ) correlation coefficients, R-squared (R^2^), p-value, and the slope (m) of the linear regression. Source data

These archaeasins were tested against clinically relevant pathogens (*Acinetobacter baumannii*, *Escherichia coli*, *Klebsiella pneumoniae*, *Pseudomonas aeruginosa*, *Staphylococcus aureus*, *Enterococcus faecalis* and *Enterococcus faecium*) at a range of concentrations from 1 μmol l^−1^ to 64 μmol l^−1^. The results showed that 75 of the 80 EPs showed antimicrobial activity (MIC ≤ 64 μmol l^−1^) against at least 1 pathogenic strain (Fig. 2a), resulting in a hit rate of over 93%. Polymyxin B and levofloxacin were used as positive controls (Extended Data Fig. 4a). In addition, the Pearson correlation (*r* = 0.503) between predicted and experimentally validated MICs showed the predictive power of APEX 1.1 (Extended Data Fig. 4b). When comparing the Pearson and Spearman correlations between experimental and predicted MICs from the first version of APEX^6^ to APEX 1.1 (used to explore the archaeome) on the 80 archaeasins synthesized, we observed that APEX 1.1 significantly outperformed APEX (Supplementary Tables 3 and 4).

### Disordered and β-rich secondary structure profiles of archaeasins

**Extended Data Fig. 5:**
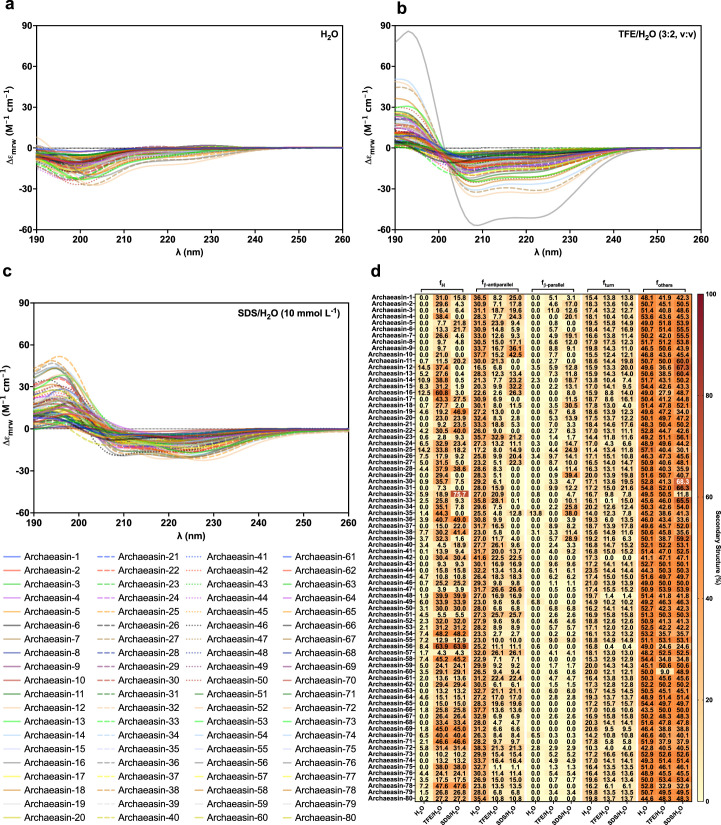
Circular dichroism spectra of archaeasins. Circular dichroism experiments were conducted with encrypted peptides from the archaeome using a J-1500 Jasco circular dichroism spectrophotometer. The spectra were recorded in three different media: **(a)** water, **(b)** 60% trifluoroethanol in water, and **(c)** sodium dodecyl sulfate (SDS) in water (10 mmol L^−1^), after three accumulations at 25 °C, using a 1 mm path length quartz cell, between 260 and 190 nm at 50 nm min^−1^, with a bandwidth of 0.5 nm. The concentration of all peptides tested was 50 μmol L^−1^. **(d)** Heatmap with the percentage of secondary structure found for each peptide in three different solvents: water, 60% trifluoroethanol (TFE) in water, and SDS (10 mmol L^−1^) in water. Secondary structure fraction was calculated using the BeStSel server^18^. Source data

The secondary structure of short peptides is often dynamic, transitioning between disordered and ordered conformations at hydrophobic–hydrophilic interfaces. These structural transitions are critical in determining the antimicrobial and other biological functions of peptides. To assess the secondary structure of the synthesized archaeasins, we conducted circular dichroism experiments in various environments: water, sodium dodecyl sulfate (SDS) in water (10 mmol l^−1^), and a mixture of trifluoroethanol (TFE) in water (3:2, v/v). SDS micelles were chosen as a membrane-mimetic environment because of the lipid bilayer environment that is similar to biological bilayers^15^. The TFE–water mixture is known to induce α-helical structures by dehydrating the amide groups in the peptide backbone, thus favouring intramolecular hydrogen bonds that promote a helical conformation^16,17^. All archaeasins were tested at 50 μmol l^−1^ in the wavelength range of 190–260 nm (Extended Data Fig. 5). To determine secondary conformation fractions, we used the Beta Structure Selection (BeStSel) server^18^ (Fig. 2b–d). As expected, given that archaeasins are short sequences (<50 amino acid residues), all tested peptides were unstructured in water (Fig. 2b), with a slight tendency towards β-like structures (20% < *f*_β_ (β fraction)< 45%) in the other 2 analysed media, the helical-inducer TFE and water mixture (3:2, v/v; Fig. 2c) and SDS micelles (10 mmol l^−1^) in water (Fig. 2d). This behaviour is typical for short peptides showing antimicrobial activity^19–21^. Whereas EPs primarily adopt β-like structures^3,8^, archaeasins showed helical conformations in helical-inducing media and upon interaction with lipid bilayers.

### Functional synergy and cooperative interactions among archaeasins

**Fig. 3:**
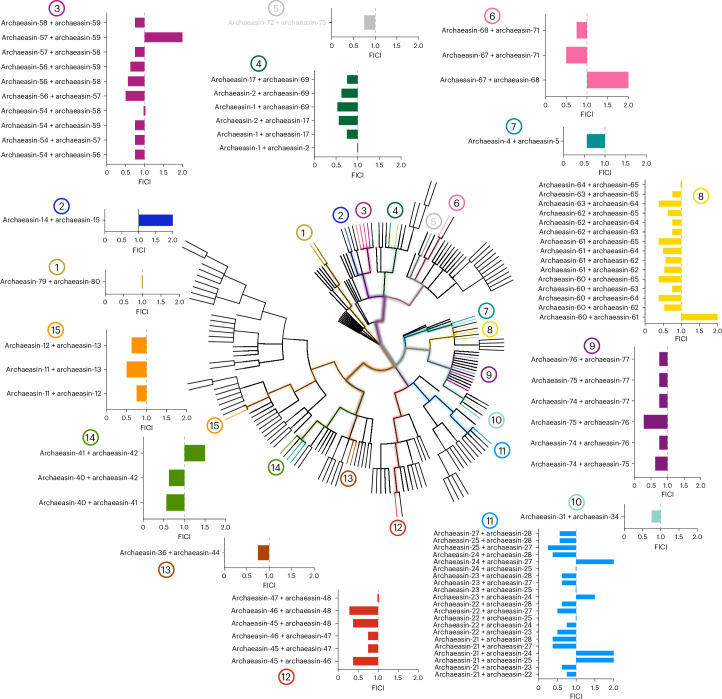
Synergistic interaction between archaeal peptide antibiotics. The synergistic interactions between pairs of EPs from the same or closely related organisms (phylogenetic pairwise distance ≤8) that showed activity against *A. baumannii* ATCC 19606 were assessed using checkerboard assays. These assays involved twofold serial dilutions, ranging from 2× MIC to a 1:32 dilution. The histogram shows the FICI values obtained for each pair of EPs. A total of 79 pairs were evaluated. Low FICI values (≤0.5) indicate synergistic interactions, intermediate values (0.5 < FICI ≤ 1) indicate additive effects and higher values (1 < FICI ≤ 2) indicate indifferent interactions. Numbers 1–15 indicate where each pair or group of pairs originates within the archaeal phylogenetic tree. Source data

To explore whether molecules from the same archaeal strains or their closest relatives could synergize and potentiate each other’s antimicrobial activity against pathogens, we performed checkerboard assays. Checkerboard assays are a standard method used to evaluate the interaction between two antimicrobial agents by testing their combined effects over a range of concentrations. This approach allows for the determination of whether a combination exhibits synergy, additivity, indifference or antagonism, providing a quantitative assessment of potential cooperative activity^22^. These assays tested peptide concentrations ranging from 2× the MIC to concentrations up to 32× lower, under the same conditions as those used for the antimicrobial assays. We initially selected the bacterial strain *A. baumannii* American Type Culture Collection (ATCC) 19606, known for its high antibiotic resistance and significant role as an opportunistic nosocomial pathogen with substantial global mortality rates^23^. This strain was particularly susceptible to the archaeasins. We then selected peptides from strains closely related on the phylogenetic tree (pairwise distance ≤8), resulting in the testing of 79 pairs of archaeasins (Fig. 3).

Most of the combinations tested showed synergistic or additive interactions, as determined by the fractional inhibitory concentration index (FICI)^24^. The FICI is commonly classified as follows: FICI ≤ 0.5 indicates strong synergy, 0.5 < FICI ≤ 1 suggests an additive effect, 1 < FICI ≤ 2 implies no interaction (indifference) and FICI > 2 denotes antagonism between the compounds. Lower FICI values indicate a stronger interaction, where the combined effect of the peptides enhances antimicrobial efficacy beyond their individual activities. Synergistic interactions are particularly significant in antimicrobial research, as they allow for lower individual drug concentrations while maintaining or enhancing efficacy. This can reduce potential toxicity, slow resistance development and improve treatment outcomes. By leveraging synergy, it may be possible to develop combination therapies that enhance the effectiveness of existing antimicrobial agents. Notably, archaeasins from *Methanocaldococcus* species showed some of the lowest FICI values, ranging from 0.25 (archaeasin-25 and archaeasin-27) to 0.375 (archaeasin-21 and archaeasin-27, archaeasin-21 and archaeasin-28, and archaeasin-24 and archaeasin-28). Similarly, *Methanothermobacter* species compounds showed FICI values from 0.28 (archaeasin-46 and archaeasin-48) to 0.375 (archaeasin-45 and archaeasin-46, and archaeasin-46 and archaeasin-48). *Thermococcus* species had a FICI of 0.28 (archaeasin-75 and archaeasin-76), while compounds derived from *Pyrococcus* species had a FICI of 0.375 for combinations of archaeasin-60 and archaeasin-64, archaeasin-60 and archaeasin-65, archaeasin-61 and archaeasin-65, and archaeasin-63 and archaeasin-64.

Interestingly, our analysis revealed that certain archaeal lineages appear to be more prone to producing synergistic peptides. Peptides from hyperthermophilic archaea, particularly those from *Methanocaldococcus* and *Thermococcus* species, showed the most consistent synergistic interactions. This observation suggests that organisms adapted to extreme environments may have evolved antimicrobial strategies that rely on cooperative mechanisms, possibly to counteract competitive microbial communities. The tendency of peptides from these lineages to show synergy could be due to their structural adaptations, enhanced stability under extreme conditions or specific physico-chemical properties that facilitate cooperative activity. Further exploration of these evolutionary trends may provide deeper insights into the origins of antimicrobial synergy and guide the design of therapeutic peptide combinations.

These findings highlight the potential for archaeasin-based combinatorial therapies to provide alternatives for antimicrobial development, particularly in addressing multidrug-resistant infections.

### Membrane-disruptive mode of action of archaeasins

**Fig. 4:**
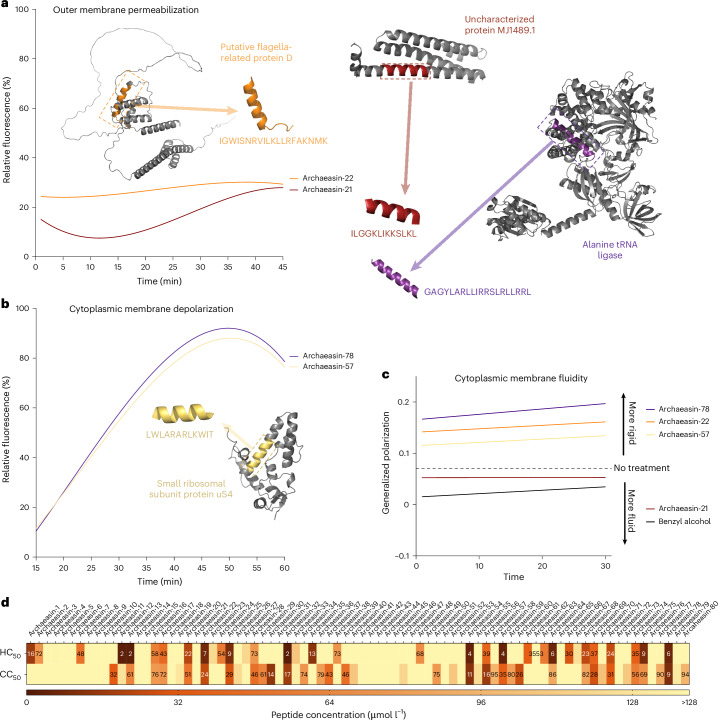
Mechanism of action, haemolytic activity and cytotoxicity of antibiotics from the archaeome. To assess whether archaea EPs act on bacterial membranes, all active peptides against *A. baumannii* ATCC 19606 were subjected to outer membrane permeabilization and cytoplasmic membrane depolarization assays. **a**,**b**, Here we show the two lead permeabilizer and depolarizer archaeasins (see Extended Data Fig. 6 for permeabilization and depolarization results of all archaeasins). The fluorescence probe NPN was used to assess membrane permeabilization (**a**) induced by the tested EPs (Extended Data Fig. 6a). The fluorescence probe DiSC_3_-5 was used to evaluate membrane depolarization (**b**) caused by archaeasins (Extended Data Fig. 6b). The shown values represent the relative fluorescence of both probes, with nonlinear fitting compared with the baseline of the untreated control (buffer + bacteria + fluorescence dye) and benchmarked against the antibiotics polymyxin B and levofloxacin. **c**, Laurdan generalized polarization over time in *A. baumannii* treated with archaeasins. Generalized polarization values were measured to assess changes in the lipid packing (membrane order) of the cytoplasmic membrane following treatment with the archaeasins that showed greater permeabilization of the outer membrane, archaeasin-21 and archaeasin-22, and greater depolarization of the cytoplasmic membrane, archaeasin-57, and archaeasin-78. Higher generalized polarization values indicate increased membrane rigidity, whereas lower values reflect increased membrane fluidity. Benzyl alcohol was used as a positive control for membrane fluidization, and untreated cells served as a negative control. Data represent a linear regression of the mean of 3 independent experiments over 30 min. **d**, Haemolytic and cytotoxic concentrations, against RBCs and HEK293T cells, respectively, leading to 50% cell lysis (HC_50_ and CC_50_, respectively) were determined by interpolating the dose-response data using a nonlinear regression curve. All experiments were performed in three independent replicates (Extended Data Fig. 7). The protein and peptide structures depicted in panels **a** and **b** were created with PyMOL Molecular Graphics System, v.3.0 (Schrödinger). Source data

**Extended Data Fig. 6:**
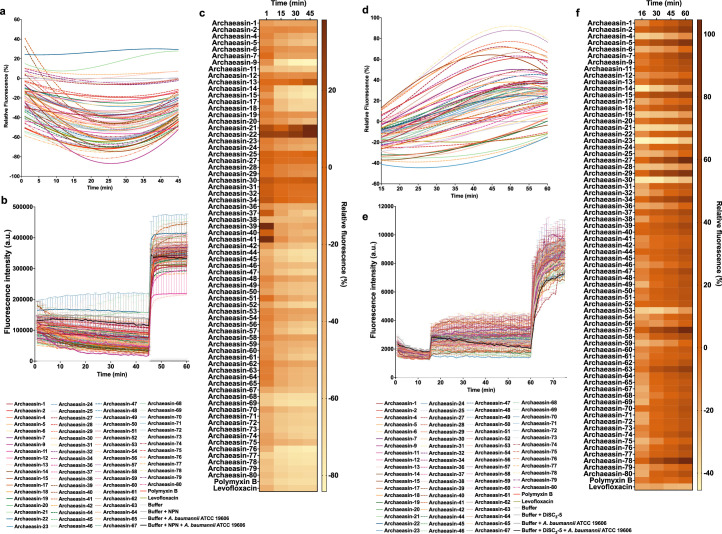
Outer membrane permeabilization and cytoplasmic membrane depolarization of *A. baumannii* ATCC 19606 induced by archaeasins. **(a-c)** Outer membrane permeabilization was assessed using the probe 1-(N-phenylamino)naphthalene (NPN), showing the permeabilization effects of Archaea-encoded encrypted peptides active against *A. baumannii* ATCC 19606: **(a)** relative fluorescence, **(b)** fluorescence intensity, and **(c)** summary of the relative fluorescence in different time points of the experiment. **(d-f)** Membrane depolarization assays were performed using the hydrophobic probe 3,3′-dipropylthiadicarbocyanine iodide [DiSC_3_-(5)] on all archaeasins active against *A. baumannii* ATCC 19606: **(d)** relative fluorescence, **(e)** fluorescence intensity, and **(f)** summary of the relative fluorescence in different time points of the experiment. Polymyxin B served as a positive control, while buffer, buffer with the probe, and buffer with both probe and bacteria were used as baseline controls for fluorescence. Error bars are the standard deviation obtained from the three replicates and relative fluorescence is shown as a non-linear regression of the fluorescence intensity normalized by the untreated control (buffer). Source data

To understand how archaeasins exert their effect on bacterial cells, we conducted fluorescence assays to determine if their mechanism of action involves membrane targeting. First, we identified 70 antimicrobial hits among archaeasins effective against *A. baumannii* ATCC 19606 (Fig. 2a). We then assessed the ability of these peptides, at their MIC values, to permeabilize (Fig. 4a and Extended Data Fig. 6a) and depolarize (Fig. 4b and Extended Data Fig. 6b) bacterial outer and cytoplasmic membranes, respectively.

To evaluate the ability of archaeasins to permeabilize the outer membrane of Gram-negative bacteria, we used N-phenyl-1-naphthylamine (NPN) assays. NPN is a lipophilic dye that fluoresces in lipid-rich environments, such as bacterial outer membranes. Damage to the bacterial outer membrane allows NPN to penetrate, increasing fluorescence (Fig. 4a). Only archaeasin-21 (parent protein, uncharacterized protein MJ1489.1) and archaeasin-22 (parent protein, putative flagella-related protein D) from *Methanocaldococcus jannaschii* effectively permeabilized the bacterial outer membrane. Polymyxin B served as a positive control in these experiments^4^. Overall, archaeasins did not permeabilize the bacterial outer membrane to the extent observed for AMPs^25,26^ or other human- or animal-derived EPs^4,6^.

We then used 3,3′-dipropylthiadicarbocyanine iodide (DiSC_3_-5), a fluorophore that indicates cytoplasmic membrane depolarization. Disruption of the transmembrane potential causes the fluorophore to migrate to the extracellular space, resulting in increased fluorescence. Among the 70 peptides tested, 34 archaeasins significantly depolarized the cytoplasmic membrane more than the control group treated with polymyxin B^4^ (Fig. 4b). Archaeasin-78 (parent protein, alanine tRNA ligase) from *Thermofilum pendens* and archaeasin-57 (parent protein, small ribosomal subunit protein uS4) from *Pyrobaculum arsenaticum* were particularly effective depolarizers.

Laurdan generalized polarization assays further supported this membrane-targeting mechanism by revealing changes in the physical state of the *A. baumannii* cytoplasmic membrane upon archaeasin treatment (Fig. 4c). Laurdan fluorescence shifts reflect alterations in membrane lipid packing, where increased generalized polarization values indicate membrane rigidification. Consistent with the cytoplasmic membrane depolarization data, archaeasin-78 and archaeasin-57 caused pronounced increases in generalized polarization values over time, suggesting that these peptides not only disrupt membrane potential but also directly perturb membrane lipid organization. In contrast, archaeasin-21 and archaeasin-22, which were effective at permeabilizing the outer membrane, induced only modest or even decreasing generalized polarization shifts, indicating minimal impact on the cytoplasmic membrane’s physical state. These differences in Laurdan response correlate with each peptide’s structural features and probable depth of membrane insertion, reinforcing the conclusion that cytoplasmic membrane interaction, rather than outer membrane permeabilization, is the dominant antimicrobial mechanism for most archaeasins.

These findings suggest that archaeasins primarily exert their antimicrobial effects by depolarizing the cytoplasmic membrane, rather than permeabilizing the outer membrane. This suggests a mechanism akin to that of the recently reported small open-reading-frame-encoded peptides^8^ and unusual for conventional AMPs^25,26^ and EPs^4^, which typically target the outer membrane^19^.

### Low toxicity of archaeasins against human cell lines

**Extended Data Fig. 7:**
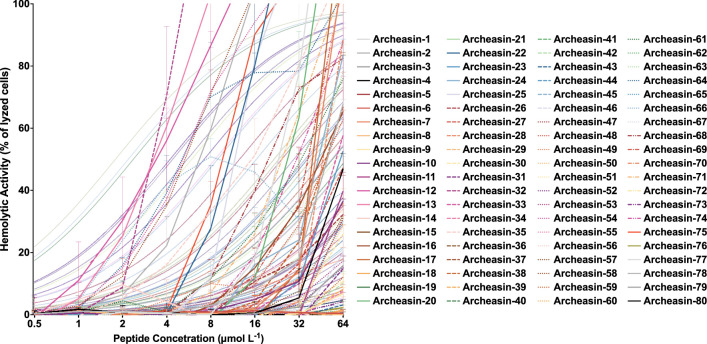
Hemolytic activity of archaeasins. Hemolytic activity was assessed by exposing red blood cells to archaeasins, with lysis determined by absorbance readings after 4 h. The peptides were added to plates and subjected to a two-fold dilution series (ranging from 64 to 4 μmol L^−1^) before exposure to the red blood cells. Source data

To assess the potential toxicity of the synthesized archaeasins, we exposed them to human red blood cells (RBCs), a common method for evaluating the toxicity of antimicrobial agents^20,26,27^. Of the 80 archaeasins tested, 25 (31.3%) showed moderate to low haemolytic activity within the explored concentration range, that is, their HC_50_ values (linear regression of the peptide concentration that leads to 50% RBC lysis) were ≤64 μmol l^−1^ (Fig. 4d and Extended Data Fig. 7). Most sequences active against bacterial pathogens at low MIC values did not display toxic effects at those concentrations (Extended Data Fig. 7). However, 7 archaeasins, specifically archaeasin-12, archaeasin-13, archaeasin-32, archaeasin-54, archaeasin-58, archaeasin-64 and archaeasin-78, did show haemolytic effects.

To further evaluate the safety profile of the archaeasins, we assessed their cytotoxic activity against human embryonic kidney (HEK293T) cells. The CC_50_ values, defined as the peptide concentration leading to 50% reduction in HEK293T cell viability, were determined for each of the 80 synthesized archaeasins (Fig. 4d). The cytotoxicity data are summarized in a heat map, indicating the concentration ranges at which cytotoxic effects were observed (Fig. 4d and Extended Data Fig. 7).

Of the 80 archaeasins tested, the majority displayed low cytotoxicity, with CC_50_ values exceeding 128 µmol l^−1^. Specifically, 26 archaeasins showed CC_50_ values higher than 128 µmol l^−1^, suggesting minimal cytotoxic effects within the tested concentration range. However, a subset of archaeasins showed low to moderate cytotoxicity. Notably, archaeasin-12, archaeasin-13, archaeasin-32, archaeasin-54, archaeasin-58, archaeasin-64 and archaeasin78, which also showed haemolytic activity, had CC_50_ values at or below 64 µmol l^−1^, indicating potential off-target toxicity.

Interestingly, most archaeasins with potent antibacterial activity (low MIC values) did not show significant cytotoxicity towards HEK293T cells at those concentrations. This selective activity highlights their potential as promising antimicrobial candidates with limited cytotoxic effects on human cells.

### Anti-infective activity of archaeasins in preclinical animal models

**Fig. 5:**
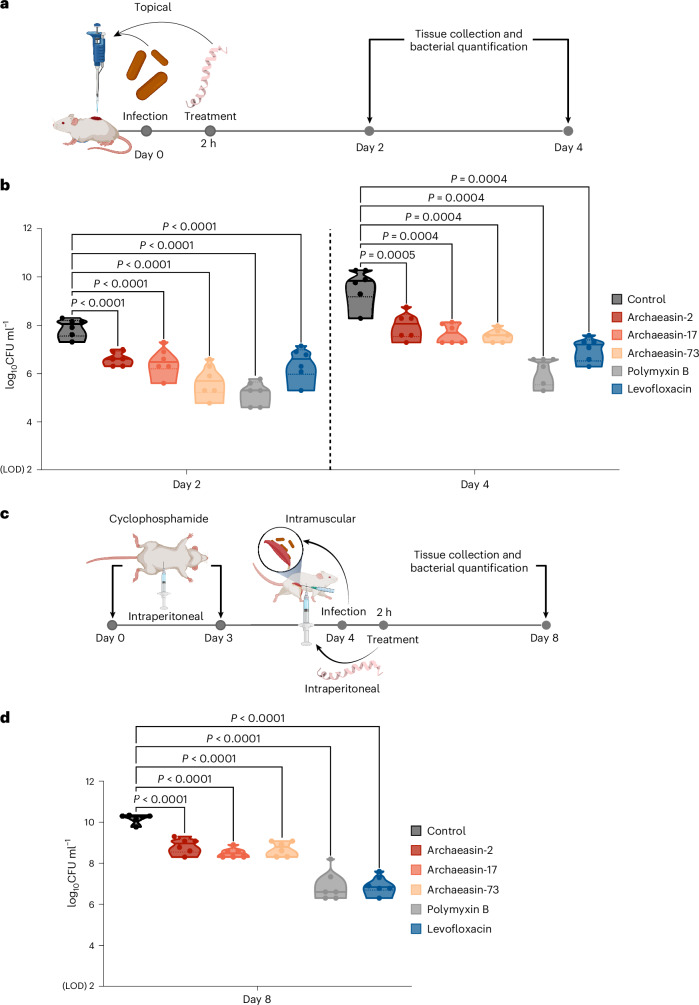
Anti-infective activity of archaeasins in animal models. **a**, Schematic representation of the skin abscess mouse model used to assess the anti-infective activity of archaeasins (*n* = 6) against *A. baumannii* ATCC 19606. **b**, Archaeasin-2, archaeasin-17 and archaeasin-73, administered at their MIC in a single dose post-infection, inhibited the proliferation of the infection for up to 4 days after treatment compared with the untreated control group. Notably, archaeasin-73 reduced the infection in some mice, showing activity comparable to the control antibiotic, polymyxin B. **c**, Schematic of the neutropenic thigh infection mouse model, where archaeasins were administered intraperitoneally. Anti-infective activity against *A. baumannii* ATCC 19606 was evaluated 4 days after intraperitoneal peptide administration (*n* = 6). **d**, At 4 days after intraperitoneal injection (day 8 of the experiment), all archaeasins at their MIC showed a bacteriostatic effect, containing the *A. baumannii* ATCC 19606 infection, although their activity was less potent than that of polymyxin B and levofloxacin, compared with the untreated control group (Extended Data Fig. 8). The limit of detection (LOD) for the CFU quantification is log_10_CFU = 2. Statistical significance in panels **b** and **d** was determined using one-way analysis of variance followed by Dunnett’s test; *P* values are shown in the graphs. In the violin, the centre line represents the mean, the box limits the 1st and 3rd quartiles, and the whiskers (minimum and maximum) represent 1.5× the interquartile range. The solid line inside each box represents the mean value obtained for each group. Panels **a** and **c** created with BioRender.com. Source data

To evaluate whether the lead archaeasins retained their antimicrobial potency in complex living systems, we tested them in two mouse models: a skin abscess model^28–30^ and a deep thigh infection model^4,5^ (Fig. 5a). In both models, we used *A. baumannii*, a pathogen responsible for infections in the blood, urinary tract, lungs, and topical wounds, and a major cause of mortality in hospitalized patients due to its antimicrobial resistance^31^. Three lead archaeasins showed potent activity against *A. baumannii* and no cytotoxicity (CC_50_ < 64 μmol l^−1^): archaeasin-2 (MIC value = 4 μmol l^−1^) from *Aeropyrum pernix*, archaeasin-17 (MIC value = 2 μmol l^−1^) from *Ignicoccus hospitalis* and archaeasin-73 (MIC value = 8 μmol l^−1^) from *Sulfurisphaera tokodaii*.

**Extended Data Fig. 8:**
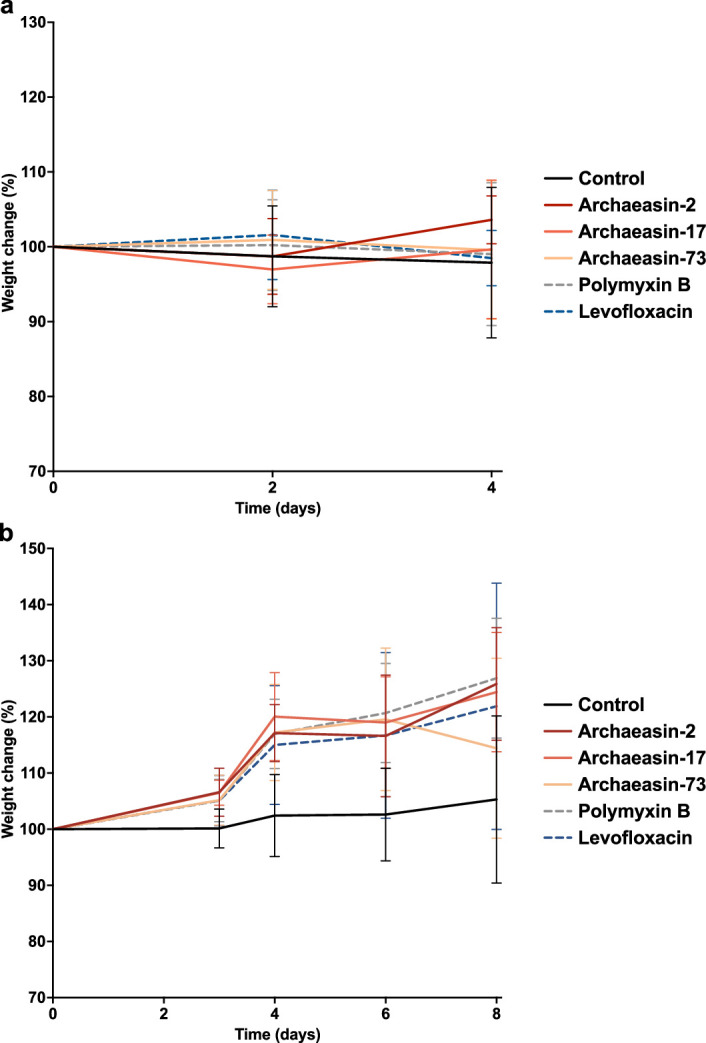
Weight change monitoring in both skin abscess and deep thigh infection mouse models infected with *A. baumannii.* Mouse weight was monitored throughout the duration of the **(a)** skin abscess model (4 days total) and the **(b)** deep thigh infection model (8 days total) to assess potential toxic effects of both the bacterial load and the archaeasins. Source data

In the skin abscess model, infection was established with a 20 μl bacterial load of 1.2 × 10^5^ *A. baumannii* cells in phosphate buffer solution (PBS) applied to a wounded area of the skin (Fig. 5a). A single dose of each archaeasin at their respective MIC was administered to the infected area. Two days post-infection, all tested archaeasins showed significantly reduced bacterial counts by 1.5 to 2 orders of magnitude. Archaeasin-73, in particular, reduced the bacterial load by two orders of magnitude compared with the untreated control group. Its potency was comparable to that observed in the positive control group of mice treated with polymyxin B and was higher than that of the levofloxacin control group (Fig. 5b). Four days post-infection, all archaeasins and the two antibiotics, polymyxin B and levofloxacin, continued to prevent bacterial growth with similar efficacy. Polymyxin B reduced bacterial counts by four orders of magnitude compared with the untreated control group of mice, while all other treatment groups showed a two- to three-order magnitude decrease. These results are promising, as the archaeasins were administered only once after the abscess had been established, highlighting their anti-infective potential. Importantly, no significant changes in weight, used as a proxy for toxicity, were observed in our experiments (Extended Data Fig. 8a).

Next, we assessed the efficacy of the same lead archaeasins (archaeasin-2, archaeasin-17 and archaeasin-73) in a murine deep thigh infection model (Fig. 5c), which is widely used to assess the antibiotic potential of compounds. Mice were administered 2 rounds of cyclophosphamide treatment for immunosuppression before the intramuscular infection with 1 × 10^5^ cells in 100 μl of *A. baumannii*. A single dose of each archaeasin (at their MIC) was delivered intraperitoneally (Fig. 5c). Four days post-treatment, the archaeasins were unable to prevent the growth of the infection, while the antibiotics polymyxin B and levofloxacin (positive controls) reduced the bacterial load by three orders of magnitude (Fig. 5d). Four days post-treatment, the bacterial counts remained stable for all peptide treatment conditions and the treatments with polymyxin B and levofloxacin, while the untreated control increased by two orders of magnitude. No significant changes in weight were observed, indicating that the archaeasins are non-toxic (Extended Data Fig. 8b). These in vivo results support the antibiotic properties of archaeasins under physiological conditions and provide a strong foundation for advancing their development as potential antimicrobial agents.

## Discussion

In this study, we systematically explored the archaeome using the deep learning model APEX 1.1, revealing a wealth of previously unrecognized antibiotic molecules within archaea. Our findings highlight the untapped potential of archaea as a source of antimicrobial agents^32–38^, expanding the traditional focus beyond bacteria and fungi, which have historically been the primary sources of antibiotics derived from nature.

We report the discovery of archaeasins, a class of AMPs with unique sequence diversity. Our synergy assays further underscored the potential of archaeasins to work in concert, enhancing their antimicrobial efficacy when combined. The low FICI values observed in combinations from closely related strains, particularly within the *Methanocaldococcus* and *Methanothermobacter* species, point to the possibility of developing combination therapies that leverage these synergistic effects. Such combinations could provide more effective treatment options, especially against multidrug-resistant pathogens.

Mechanism-of-action studies revealed that archaeasins primarily exert their antimicrobial effects by depolarizing the bacterial cytoplasmic membrane, rather than by permeabilizing the outer membrane. This finding is particularly intriguing, as it suggests that archaeasins may operate through a mechanism distinct from that of conventional AMPs, which often target the outer membrane. Depolarization of the cytoplasmic membrane is a critical process that disrupts bacterial homeostasis, leading to cell death. Interestingly, this mode of action aligns more closely with that of recently described small open-reading-frame-encoded peptides^8^.

Our in vivo experiments in mouse models showed that archaeasins retain their antimicrobial potency in complex biological systems, effectively reducing bacterial loads in both skin abscess and deep thigh infection models. The observed efficacy, particularly with archaeasin-73, which showed results comparable to that of traditional antibiotics like polymyxin B and levofloxacin, is promising. These results suggest that archaeasins have the potential to be developed into viable therapeutic agents, especially for infections caused by multidrug-resistant pathogens such as *A. baumannii*. Importantly, the lack of significant toxicity observed in these models further supports the safety profile of these peptides, a crucial consideration for future development.

Despite the promising results, several challenges and limitations remain. For instance, although the in vivo findings are encouraging, further studies are necessary to systematically evaluate the long-term efficacy and safety of archaeasins, including their pharmacokinetics, pharmacodynamics and potential immunogenicity in humans.

In addition, there are inherent limitations in using deep learning to explore archaeal proteomes as a framework for antibiotic discovery. Our current deep learning model is sequence based and lacks structural information. Although this approach allows for rapid analysis across proteomes, incorporating structural and three-dimensional descriptors in future iterations could improve the model’s accuracy in predicting antimicrobial activity. To prevent APEX from making over-optimistic MIC predictions, we included ‘inactive’ data points (that is, data with MICs above the maximum concentrations tested) in the model training and assigned a pseudo-MIC label of 512 μmol l^−1^ to them. However, the pseudo-MIC labels may not perfectly reflect the true MICs of these points, potentially introducing noise into the model. A future direction will be to use contrastive loss during model training, eliminating the need for concrete assumptions about the MICs of inactive data. Another challenge is the limited availability of information on archaeal proteins. The virtual screening of archaea proteomes was performed only on the high-quality reviewed sequences from UniProt, while the unreviewed sequences were not included in the analysis. This inevitably led to an imbalanced analysis, with some interesting clades (for example, DPANN and *Asgardarchaeota*) not being well characterized by APEX. In the future, we will apply APEX to screen unreviewed archaea proteins to complement this study.

In this study, we used only female mice to maintain consistency with established protocols and previous studies^4,6,20,29,30,39,40^, ensuring better comparability of results. However, sex-based physiological variations, including differences in immune response, hormone levels and microbiota composition, may influence infection outcomes and antimicrobial efficacy.

Another limitation is the observed decrease in efficacy of archaeasins between days 2 and 4 in the skin infection model, probably due to its susceptibility to proteolytic degradation and clearance in vivo. Addressing this challenge will require future studies to explore peptide stabilization strategies, such as chemical modifications (for example, d-amino acid incorporation, cyclization or PEGylation), to enhance in vivo stability and extend their therapeutic efficacy.

In addition, although EPs have been shown to be less likely to promote bacterial resistance than conventional antibiotics^4,30^, future studies will evaluate the potential for archaeasins to induce resistance.

A comparison with random sequences matching organismal amino acid distributions would shed additional light on how sequence composition influences antimicrobial potential. In our previous work^3^, scrambled versions of EPs from bacteria, which no longer reflect the natural arrangement of amino acids, were inactive. This result suggests that the biologically derived sequences are essential for the antimicrobial effect. Furthermore, we^4,7,41,42^ and others^43^ have reported the discovery of bioactive ‘hidden’ or ‘encrypted’ peptides in proteomes. Altogether, this work^38^ supports our hypothesis^7^ that many previously unrecognized bioactive peptides, including those derived from the human proteome^7^, can play critical roles in host immunity and other physiological processes^7^.

In conclusion, our study shows the promise of using deep learning to unlock the archaeome as a source of antibiotics. The discovery of archaeasins warrants further development of these agents, opening a new frontier in the fight against antimicrobial resistance.

## Methods

### Peptides in archaeal proteomes

All reviewed canonical and isoform sequences of archaea (taxon identifier, 2157) were downloaded from UniProt (https://www.uniprot.org/; access date, 24 August 2023). We were able to obtain 19,710 protein sequences (18,677 non-redundant sequences) from 233 archaeal organisms. Protein substrings ranging from 8 to 50 amino acid residues in the 18,677 sequences and containing only canonical amino acids were considered as the archaea EPs. In total, we obtained 193,331,608 EPs from the archaeome for further study.

### APEX 1.1

APEX is a bacterial-strain-specific antimicrobial activity predictor^6^, and was trained on an in-house peptide dataset and publicly available AMPs. Here we updated the training data and retrained APEX (that is, APEX 1.1). Specifically, the updated in-house peptide dataset for training APEX contained 15,718 MIC values from 1,642 peptides and 11 pathogenic strains (*A. baumannii* ATCC 19606, *E. coli* ATCC 11775, *E. coli* AIC221, *E. coli* AIC222, *K. pneumoniae* ATCC 13883, *P. aeruginosa* PAO1, *P. aeruginosa* PA14, *S. aureus* ATCC 12600, methicillin-resistant *S. aureus* ATCC BAA-1556, vancomycin-resistant *E. faecalis* ATCC 700802 and vancomycin-resistant *E. faecium* ATCC 700221). Inactive data points, that is, MIC values higher than maximum concentrations tested, were labelled as 512 μmol l^−1^. All MICs were then transformed by $-{\log }_{10}\frac{{\rm{MIC}}\;{\rm{value}}}{1,000,000}$. In addition to the in-house data, we curated 19,564 publicly available AMPs from DBAASP^11^, APD3 (ref. ^12^) and DRAMP^13^ that did not overlap with our in-house data, and 9,857 non-AMPs following the instructions from refs. ^44,45^. These publicly available AMPs and non-AMPs were used as a data augmentation strategy during APEX training^6^. We followed the original APEX paper^6^ for hyperparameter selection. The top eight APEX models were selected to create an ensemble learning where the final MIC prediction was defined as the mean predictions of the selected models. The training of APEX 1.1 was conducted using PyTorch (v.1.11.0+cu113).

### Physico-chemical properties analysis

The six physico-chemical properties of peptides, including normalized hydrophobic moment, normalized hydrophobicity, net charge, disordered conformation propensity, propensity to aggregation in vitro and amphiphilicity index, were obtained from the DBAASP server^11^. Note that the Eisenberg and Weiss scale^46^ was chosen as the hydrophobicity scale.

### Phylogenetic tree visualization and phylogenetic distance

To obtain the phylogenetic tree, the taxon identifiers of 233 archaeal organisms obtained from UniProt were uploaded to NCBI Taxonomy Common Tree (https://www.ncbi.nlm.nih.gov/Taxonomy/CommonTree/wwwcmt.cgi). Of note, 201 of 233 archaea organisms were successfully retrieved. The resulted tree file from NCBI was then visualized via iTOL (https://itol.embl.de/). The distance of two nodes in the phylogenetic tree is defined as the length of the shortest path between these two nodes.

### Peptide sequence similarity

Let SW(*i*, *j*) denote the Smith–Waterman alignment score^47^ between two protein sequences *i* and *j*. We define the peptide sequence similarity between *i* and *j* as $\frac{{\rm{SW}}(i,\;j)}{\sqrt{{\rm{SW}}\left(i,i\right){\rm{SW}}(j,\;j)}}$.

### Peptide sequence space visualization

Given a peptide dataset, we calculated a similarity matrix to represent the pairwise sequence similarities among the peptides. We then applied UMAP to transform this similarity matrix into a two-dimensional space. This transformed space serves as a proxy for the peptide sequence space, allowing us to visualize the distribution of peptides within it.

### Archaeasin selection

APEX 1.1 was used to predict the antimicrobial activity for the 193,331,608 EPs derived from the archaeome. We used the mean MIC value against the 11 pathogen strains to rank and select the EPs for chemical synthesis and experimental validation (Supplementary Data 2). When selecting the peptides, we also made sure they met the following criteria:

1. The selected peptide should have <70% sequence similarity to all in-house peptides and publicly available AMPs.
2. The selected peptides themselves should have <70% sequence similarity.
3. The selected peptide should have ≤80 μmol l^−1^ mean MIC by prediction (there are 265 peptides that meet all three criteria, so we selected the synthesized peptides from the top 265 scored peptides from the 193,331,608 screened ones).

To facilitate chemical synthesis and minimize undesired side products, we excluded from the APEX selection all peptides containing more than one cysteine residue.

Peptides with multiple cysteines often require specialized conditions for disulfide bond formation and may introduce structural heterogeneity that complicates downstream validation.

Peptide sequences with high potential for aggregation (motifs known to cause aggregation and hydrophobic clusters) were also excluded from the selection.

### Peptide synthesis

All peptides used in the experiments were purchased from AAPPTec and synthesized by solid-phase peptide synthesis using the 9-fluorenylmethyloxycarbonyl (Fmoc) strategy.

### Culturing conditions and bacterial strains

In this study, we used the following pathogenic bacterial strains obtained from the ATCC: *A. baumannii* ATCC 19606, *E. coli* ATCC 11775, *K. pneumoniae* ATCC 13883, *P. aeruginosa* PAO1, *P. aeruginosa* PA14, *S. aureus* ATCC 12600, *S. aureus* ATCC BAA-1556 (methicillin-resistant strain), *E. faecalis* ATCC 700802 (vancomycin-resistant strain) and *E. faecium* ATCC 700221 (vancomycin-resistant strain). *E. coli* AIC221 (*E. coli* MG1655 phnE_2::FRT; control strain for AIC222) and *E. coli* AIC222 (*E. coli* MG1655 pmrA53 phnE_2::FRT; polymyxin resistant, colistin-resistant strain) were kindly donated by Prof. Mark Goulian (University of Pennsylvania). Pseudomonas Isolation (*P. aeruginosa* strains) agar plates were exclusively used in the case of *Pseudomonas* species. All other pathogens were grown in Luria-Bertani (LB) broth and on LB agar. In all experiments, bacteria were inoculated from 1 isolated colony and grown overnight (16 h) in liquid medium at 37 °C. On the following day, inoculums were diluted 1:100 in fresh media and incubated at 37 °C to mid-logarithmic phase.

### Human cells and serum

HEK293T cells were obtained from ATCC (CRL-3216). RBCs and human serum were purchased from Zen-Bio. The RBC samples were obtained from the same certified healthy donor (blood type A^−^).

### MIC determination

Broth microdilution assays were performed to determine the MIC values of each peptide. Peptides were added to non-treated polystyrene microtitre 96-well plates and 2-fold serially diluted in sterile water from 1 μmol l^−1^ to 64 μmol l^−1^. Bacterial inoculum at 4 × 10^6^ colony-forming units (CFU) per ml in LB medium was mixed 1:1 with the peptide. The MIC was defined as the lowest concentration of peptide able to completely inhibit the bacterial growth after 24 h of incubation at 37 °C. All assays were done in three independent replicates.

### Circular dichroism experiments

The circular dichroism experiments were conducted using a J1500 circular dichroism spectropolarimeter (Jasco) in the Biological Chemistry Resource Center (BCRC) at the University of Pennsylvania. Experiments were performed at 25 °C, the spectra graphed are an average of 3 accumulations obtained with a quartz cuvette with an optical path length of 1.0 mm, ranging from 190 nm to 260 nm at a rate of 50 nm min^−^^1^ and a bandwidth of 0.5 nm. The concentration of all peptides tested was 50 μmol l^−1^, and the measurements were performed in water, a mixture of TFE and water in a 3:2 ratio, and SDS in water at 10 mmol l^−1^, with respective baselines recorded before measurement. A Fourier transform filter was applied to minimize background effects. Secondary structure fraction values were calculated using the single spectra analysis tool on the server BeStSel^18^. Ternary plots^48,49^ were created in https://www.ternaryplot.com/ and subsequently edited.

### Synergy assays

Combinations of two EPs from the same protein were tested against *A. baumannii* ATCC 19606 strains using the checkerboard assay. Briefly, 2-fold serial dilutions of each peptide were orthogonally mixed and incubated with a bacterial suspension at a final concentration of 2 × 10^6^ CFU ml^−1^ in LB for 24 h at 37 °C. The FICIs were defined to attribute whether the interactions between peptides were synergistic (FICI ≤ 0.5), additive (0.5 > FICI ≥ 1) or indifferent (FICI > 1), and were calculated using the following equation:

$${\rm{FICI}}=\frac{{\rm{MIC}}_{A,{\rm{new}}}}{{\rm{MIC}}_{A,{\rm{old}}}}+\frac{{\rm{MIC}}_{B,{\rm{new}}}}{{\rm{MIC}}_{B,{\rm{old}}}}$$

where *A* and *B* are the two peptides, old MIC represents the MIC values obtained for the standalone peptides, and new MIC values are defined by the MIC values obtained for the combination of peptides, considering the checkerboard assay.

### Outer membrane permeabilization assays

The NPN uptake assay was used to evaluate the ability of the peptides to permeabilize the bacterial outer membrane. Inocula of *A. baumannii* ATCC 19606 were grown to an optical density (OD) at 600 nm of 0.4, centrifuged (9,391*g* at 4 °C for 10 min), washed and resuspended in 5 mmol l^−1^ HEPES buffer (pH 7.4) containing 5 mmol l^−1^ glucose. The bacterial solution was added to a white 96-well plate (100 μl per well) together with 4 μl of NPN at 0.5 mmol l^−1^. Peptides diluted in water were then added to each well, and fluorescence was measured at an excitation wavelength (λ_ex_) of 350 nm and an emission wavelength (λ_em_) of 420 nm for 45 min. The relative fluorescence was calculated using the untreated control (buffer + bacteria + fluorescence dye) and polymyxin B (positive control) as baselines, and the following equation was applied to reflect the percentage of difference between the baselines and the sample:

$$\begin{array}{l}\rm{Percentage}\; \rm{difference}\\=\frac{100 ({\rm{fluorescence}}_{\rm{sample}}-{\rm{fluorescence}}_{\rm{untreated}\; \rm{control}})}{{\rm{fluorescence}}_{\rm{untreated}\; \rm{control}}}\end{array}$$

### Cytoplasmic membrane depolarization assays

The cytoplasmic membrane depolarization assay was performed using the membrane-potential-sensitive dye DiSC3-5. *A. baumannii* ATCC 19606 and *P. aeruginosa* PAO1 in the mid-logarithmic phase were washed and resuspended at an OD of 0.05 (optical density value at 600 nm) in HEPES buffer (pH 7.2) containing 20 mmol l^−1^ glucose and 0.1 mol l^−1^ KCl. DiSC3-5 at 20 μmol l^−1^ was added to the bacterial suspension (100 μl per well) for 15 min to stabilize the fluorescence, which indicates the incorporation of the dye into the bacterial membrane. Peptides were then mixed 1:1 with the bacteria to a final concentration corresponding to their minimal inhibitory concentration needed to kill 100% of the bacterial cells (MIC_100_) values. Membrane depolarization was then followed by reading changes in the fluorescence (λ_ex_ = 622 nm, λ_em_ = 670 nm) over time for 60 min. The relative fluorescence was calculated using the untreated control (buffer + bacteria + fluorescence dye) and polymyxin B (positive control) as baselines, and the following equation was applied to reflect the percentage of difference between the baselines and the sample:

$$\begin{array}{l}\rm{Percentage}\; \rm{difference}\\=\frac{100 ({\rm{fluorescence}}_{\rm{sample}}-{\rm{fluorescence}}_{\rm{untreated}\; \rm{control}})}{{\rm{fluorescence}}_{\rm{untreated}\; \rm{control}}}\end{array}$$

### Cytoplasmic membrane fluidity assays with Laurdan

Cytoplasmic membrane order in *A. baumannii* was assessed using the generalized polarization of Laurdan fluorescence. Overnight cultures were prepared in LB broth and subcultured 1:100 into fresh PBS supplemented with 0.1–0.2% glucose. Cultures were grown at 37 °C with shaking (21*g*) until mid-log phase (OD_600_ ≈ 0.5). Bacterial cells were pelleted, washed 2× and resuspended in Laurdan buffer (PBS with 0.1–0.2% glucose and 1% dimethylformamide) to an OD_600_ of 0.5. Laurdan (10 μmol l^−1^ final concentration from a 0.5 mmol l^−1^ stock in dimethylformamide) was added, and cells were incubated for 10 min at 30 °C in the dark. After washing, 200 μl aliquots were transferred in triplicate into a black 96-well plate. Peptides (at 100× MIC) or benzyl alcohol (positive control; 5 mmol l^−1^ final concentration) were added (2 μl per well; 1:100 dilution), and fluorescence was monitored over 30 min in 1-min intervals using a plate reader (λ_ex_ = 350 nm, λ_em_ = 440 nm and 490 nm). Controls included untreated cells, buffer with Laurdan only and Laurdan-stained cells without peptide treatment. Generalized polarization was calculated as:

$$\rm{Generalized}\; \rm{polarization}=\frac{({\rm{fluorescence}}_{440\;\rm{nm}}-{\rm{fluorescence}}_{490\;\rm{nm}})}{(\rm{fluorescence}_{440\;\rm{nm}}-{\rm{fluorescence}}_{490\;\rm{nm}})}$$

### Haemolytic activity assays

To evaluate the release of haemoglobin from human erythrocytes upon treatment of each of the EPs, human RBCs were obtained from Zen-Bio (male donor, blood type A^−^) obtained from heparin anti-coagulated blood. RBCs were washed with PBS (pH 7.4) 4× by centrifugation at 800*g* for 10 min. Aliquots of 200-fold-diluted cells (75 μl) were mixed with peptide solution (0.78–100 μmol l^−1^; 75 μl), and the mixture was incubated for 4 h at room temperature. After incubation, the plate was centrifuged at 1,300*g* for 10 min to precipitate cells and debris, and 100 μl of supernatant from each well was transferred to a new 96-well plate for absorbance reading (405 nm) using an automatic plate reader. The percentage of haemolysis was defined by comparison with negative control (samples containing PBS) and positive control (samples containing 1% (v/v) SDS in PBS solution).

$$\begin{array}{l}\rm{Haemolysis}\;( \% )\\=\displaystyle\frac{100{\rm{\times }}({{absorbance}}_{405\;{nm\; peptide}}-{{absorbance}}_{405\;{nm\; negative\; control}})}{({absorbance}_{405\;{nm\; positive\; control}}-{{absorbance}}_{405\;{nm\; negative\; control}})}\end{array}$$

### Cytotoxicity assays

The cells were cultured in high-glucose Dulbecco’s modified Eagle’s medium supplemented with 1% penicillin and streptomycin (antibiotics) and 10% fetal bovine serum and grown at 37 °C in a humidified atmosphere containing 5% CO_2_.

One day before the experiment, 100 μl aliquots of HEK293T cells, at a concentration of 50,000 cells ml^−1^, were seeded into each well of 96-well plates (5,000 cells per well). Following cell attachment, the HEK293T cells were treated with increasing concentrations of peptides (ranging from 8 μmol l^−1^ to 128 μmol l^−1^) and incubated for 24 h. After the exposure period, cytotoxicity was assessed using the 3-(4,5-dimethylthiazol-2-yl)-2,5-diphenyltetrazolium bromide (MTT) assay. Specifically, the MTT reagent was prepared at a concentration of 0.5 mg ml^−1^ in phenol red-free medium and used to replace the peptide-containing supernatants (100 μl per well). The plates were then incubated for 4 h at 37 °C in a humidified atmosphere with 5% CO_2_, facilitating the formation of insoluble formazan crystals. These crystals were subsequently dissolved in 0.04 mol l^−^^1^ hydrochloric acid prepared in anhydrous isopropanol. Absorbance was measured at 570 nm using a spectrophotometer to quantify cell viability. All experiments were conducted in triplicate (three biological replicates).

### Skin abscess infection mouse model

The backs of 6-week-old female CD-1 mice under anaesthesia were shaved and injured with a superficial linear skin abrasion made with a needle. An aliquot of *A. baumannii* ATCC 19606 (6.3 × 10^5^ CFU ml^−1^; 20 μl) previously grown in LB medium to an OD of 0.5 (optical value at 600 nm) and then washed 2× with sterile PBS (pH 7.4, 9,391*g* for 3 min) was added to the scratched area. Peptides diluted in sterile water at their MIC value were administered to the wounded area 1 h post-infection. At 2 and 4 days post-infection, animals were killed, and a uniform excision of the scarified skin was excised, homogenized using a bead beater (25 Hz for 20 min), 10-fold serially diluted and plated on McConkey agar plates for CFU quantification. The experiments were performed using six mice per group. Mice were single-housed to avoid cross-contamination and maintained under a 12-h light/12-h dark cycle at 22 °C with humidity controlled at 50%. The skin abscess infection mouse model was revised and approved by the University Laboratory Animal Resources from the University of Pennsylvania (protocol 806763).

### Deep thigh infection mouse model

Experiments were performed using 6-week-old female CD-1 mice, which were rendered neutropenic by intraperitoneal application of 2 doses of cyclophosphamide (150 mg kg^−1^ and 100 mg kg^−1^) at 3 and 1 days before the infection. At day 4 of the experiment, the mice were infected in their right thigh through a 100 μl intramuscular injection of *A. baumannii* ATCC19606 (in PBS at a concentration of 1 × 10^6^ CFU ml^−^^1^). The bacterial cells were grown in LB broth, washed twice with PBS solution and diluted at the desired concentration before infecting the mice. The peptides were administered intraperitoneally 2 h after the infection. At 4 days post-infection, mice were killed and a uniform excision of the tissue from the right thigh was excised, homogenized using a bead beater (25 Hz for 20 min), 10-fold serially diluted and plated on McConkey agar plates for bacterial colony counting. The experiments were performed using six mice per group. Mice were housed in groups of 3 and maintained under a 12-h light/12-h dark cycle at 22 °C with humidity controlled at 50%. The deep thigh infection mouse model was revised and approved by the University Laboratory Animal Resources from the University of Pennsylvania (protocol 807055).

### Reproducibility of the experimental assays

All assays were performed in three independent biological replicates as indicated in each figure legend and in the Methods. The values obtained for haemolytic activity were estimated by nonlinear regression based on the screen of peptides in a gradient of concentrations and represent the haemolytic concentration values needed to lyse and kill 50% of the cells present in the experiment. In the skin abscess and thigh infection mouse models, we used six mice per group following established protocols approved by the University Laboratory of Animal Resources of the University of Pennsylvania.

### Quantification and statistical analysis

In the mouse experiments, all the raw data were log_10_-transformed and statistical significance was determined using one-way analysis of variance followed by Dunnett’s test. All the *P* values are shown for each of the groups, and all groups were compared with the untreated control group. All calculation and statistical analyses of the experimental data were conducted using GraphPad Prism v.10.3. Statistical significance between different groups was calculated using the tests indicated in each figure legend. No statistical methods were used to predetermine sample size.

### Reporting summary

Further information on research design is available in the Nature Portfolio Reporting Summary linked to this article.

## Supplementary information

Supplementary Information Supplementary Tables 1–4.

Reporting Summary

Supplementary Data 1 List of 12,632 archaeasin sequences found by APEX with predicted MICs.

Supplementary Data 2 Archaeasins predicted active with source organisms.

Supplementary Data 3 Amino acid percentage Mann–Whitney one-side greater *P* value table.

Supplementary Data 4 Amino acid percentage Mann–Whitney one-side lower *P* value table.

## Source data

Source Data Fig. 1 Statistical source data.

Source Data Fig. 2 Statistical source data.

Source Data Fig. 3 Statistical source data.

Source Data Fig. 4 Statistical source data.

Source Data Fig. 5 Statistical source data.

Source Data Extended Data Fig. 2 Statistical source data.

Source Data Extended Data Fig. 3 Statistical source data.

Source Data Extended Data Fig. 4 Statistical source data.

Source Data Extended Data Fig. 5 Statistical source data.

Source Data Extended Data Fig. 6 Statistical source data.

Source Data Extended Data Fig. 7 Statistical source data.

Source Data Extended Data Fig. 8 Statistical source data.

## Data Availability

This study did not generate new unique reagents. All reviewed canonical and isoform sequences of archaea (taxon identifier, 2157) can be downloaded from UniProt (https://www.uniprot.org/). The AMPs analysed in this study were obtained from publicly available databases, including DBAASP^11^ (https://dbaasp.org/home), APD3 (ref. ^12^) (https://aps.unmc.edu/) and DRAMP^13^ (http://dramp.cpu-bioinfor.org/). Supplementary Data 1–4, gene ontology analysis of all archaeasins validated, and their certificate of analysis are also accessible via Mendeley Data (https://data.mendeley.com/datasets/d8yzgtdrcp/3). Further information and requests for resources should be directed to the corresponding author. Source data are provided with this paper.

## Code availability

APEX 1.1 is available at GitLab (https://gitlab.com/machine-biology-group-public/apex-pathogen).
