# Supplementary material for: Deep learning reveals antibiotics in the archaeal proteome
Source: Nat Microbiol. 2025 Aug 12;10(9):2153–67. doi: 10.1038/s41564-025-02061-0 (PMC12408343; doi:10.1038/s41564-025-02061-0)
Supplement: Supplementary file 1 — Supplementary Tables 1–4. [file 41564_2025_2061_MOESM1_ESM.pdf]

nature microbiology

Supplementary information https://doi.org/10.1038/s41564-025-02061-0

Deep learning reveals antibiotics in the archaeal proteome

In the format provided by the authors and unedited

Supplementary Materials for

Deep learning reveals antibiotics in the archaeal proteome

Marcelo D. T. Torres [1-4, †], Fangping Wan [1-4, †], and Cesar de la Fuente-Nunez [1-4, *]

The PDF file includes:

Supplementary Tables 1 to 4

Supplemental tables

Supplementary Table 1. Number of predicted active archaeasins and number of genes on NCBI for selected genera. The total number of predicted active archaeasins and the number of protein-coding genes (from NCBI) for the 10 archaeal genera with the highest and lowest active EP counts.

Genus	Number of active EPs	Number of genes on NCBI	Active EPs per gene
Pyrococcus	2,184	20,225	0.108
Methanocaldococcus	1,822	12,856	0.142
Pyrobaculum	1,749	19,067	0.092
Sulfolobus	1,181	38,170	0.031
Thermococcus	881	94,875	0.009
Methanococcus	636	26,682	0.024
Archaeoglobus	541	13,166	0.041
Aeropyrum	460	3,888	0.118
Saccharolobus	442	120,015	0.004
Staphylothermus	412	3,638	0.113
Methanomethylophilus	0	5,433	0.000
Haloplanus	0	37,874	0.000
Halococcus	0	6,909	0.000
Methanogenium	0	5,025	0.000
Methanolobus	0	34,979	0.000
Methanomassiliicoccus	0	4,073	0.000
Candidatus Lokiarchaeum	0	5,337	0.000
Acidilobus	0	3,323	0.000
Halogeometricum	0	11,451	0.000
Pyrodictium	0	4,639	0.000

Supplementary Table 2. Effect sizes for the analysis of physicochemical properties across different peptide group pairs. Cohen's d effect size was calculated for each pairwise comparison, indicating that most differences were biologically meaningful. General guidelines: small (d = 0.2), medium (d = 0.5), and large (d = 0.8).

**Physicochemical property**	**DB vs. APEX (HP)**	**DB vs. SF (HP)**	**DB vs. Archaeasins**	**APEX (HP) vs. SF (HP)**	**APEX (HP) vs. Archaeasins**	**SF (HP) vs. Archaeasins**
Angle Subtended by the Hydrophobic Residues	0.196	0.688	0.853	0.403	0.598	0.310
Net charge	-0.240	-1.295	-1.489	-1.263	-1.663	-0.327
Propensity to PPII coil	-0.563	-0.937	-0.529	-0.749	0.369	0.792
Linear Moment	0.0968	0.054	0.992	-0.043	0.894	0.965
Tilt Angle	-0.130	0.002	-0.129	0.130	0.007	-0.124
Penetration Depth	0.234	0.550	-0.426	0.301	-0.687	-1.038
Amphiphilicity Ind	0.346	0.148	-0.133	-0.236	-0.672	-0.448
Propensity to In Vivo Agg	0.814	0.656	0.4715	-0.363	-0.204	0.178
Disorderd Conformation Propensity	-0.324	-0.192	0.331	0.158	0.701	0.638
Normalized Hydrophobic Moment	0.435	0.523	0.465	0.147	0.104	-0.045
Normalized Hydrophobicity	0.326	0.106	-0.311	-0.245	-0.673	-0.497
Isoelectric Point	-0.748	-1.278	-0.863	-0.818	-0.573	0.301

DB: databases; HP: human proteome; SF: scoring function.

Supplementary Table 3. Pearson correlation between predicted and experimentally-determined MICs by APEX and APEX 1.1 on the 80 Archaea encrypted peptides we synthesized and validated.

**Bacterial strains**	**APEX1.1**	**APEX**
A. baumannii ATCC 19606	0.3951	0.2038
E. coli ATCC 11775	0.2488	0.1201
E. coli AIC221	0.2553	0.0760
E. coli AIC222	0.1886	0.1253
K. pneumoniae ATCC 13883	0.1020	-0.0394
P. aeruginosa PAO1	0.3037	0.3657
P. aeruginosa PA14	0.4662	0.3608
S. aureus ATCC 12600	0.3213	0.1622
Methicillin-resistant *S. aureus* ATCC BAA-1556	0.1029	0.0698
Vancomycin-resistant *E. faecalis* ATCC 700802	0.1772	0.0824
Vancomycin-resistant *E. faecium* ATCC 700221	0.3634	0.2960
Macro average	0.2659	0.1657
Micro average	0.5033	0.4455

Supplementary Table 4. Spearman correlation between predicted and experimentally-determined MICs by APEX and APEX 1.1 on the 80 Archaea encrypted peptides we synthesized and validated.

Bacterial strains	APEX1.1	APEX
A. baumannii ATCC 19606	0.3971	0.2142
E. coli ATCC 11775	0.2282	0.1328
E. coli AIC221	0.2405	0.0707
E. coli AIC222	0.1944	0.0935
K. pneumoniae ATCC 13883	0.1314	-0.0559
P. aeruginosa PAO1	0.2882	0.3380
P. aeruginosa PA14	0.4932	0.3906
S. aureus ATCC 12600	0.2575	0.1442
Methicillin-resistant *S. aureus* ATCC BAA-1556	0.0122	0.0544
Vancomycin-resistant *E. faecalis* ATCC 700802	0.1785	0.1539
Vancomycin-resistant *E. faecium* ATCC 700221	0.3242	0.2330
Macro average	0.2496	0.1609
Micro average	0.4908	0.4340
